# Chromosome Pairing in Hybrid Progeny between *Triticum aestivum* and *Elytrigia elongata*

**DOI:** 10.3389/fpls.2017.02161

**Published:** 2017-12-19

**Authors:** Fang He, Piyi Xing, Yinguang Bao, Mingjian Ren, Shubing Liu, Yuhai Wang, Xingfeng Li, Honggang Wang

**Affiliations:** ^1^Guizhou Subcenter of National Wheat Improvement Center, College of Agronomy, Guizhou University, Guiyang, China; ^2^State Key Laboratory of Crop Biology, Shandong Key Laboratory of Crop Biology, College of Agronomy, Shandong Agricultural University, Taian, China; ^3^College of Life Science, Zaozhuang University, Zaozhuang, China

**Keywords:** *E. elongata*, *T. aestivum*, chromosome pairing, hybrid progenies, genomic *in situ* hybridization

## Abstract

In this study, the intergeneric hybrids F_1_, F_2_, BC_1_F_1_, BC_1_F_2_, and BC_2_F_1_ from *Elytrigia elongata* and *Triticum aestivum* crosses were produced to study their chromosome pairing behavior. The average *E. elongata* chromosome configuration of the two F_1_ hybrids agreed with the theoretical chromosome configuration of 21I+7II, indicating that the genomic constitution of this F_1_ hybrid was ABDStStE^e^E^b^E^x^. Compared with the BC_1_F_1_ generation, the BC_2_F_1_ generation showed a rapid decrease in the number of *E. elongata* chromosomes and the BC_1_F_2_ generation showed a more extensive distribution of *E. elongata* chromosomes. In addition, pairing between wheat and *E. elongata* chromosomes was detected in each of the wheat*-E. elongata* hybrid progenies, albeit rarely. Our results demonstrated that genomic *in situ* hybridization (GISH) using an *E. elongata* genomic DNA probe offers a reliable approach for characterizing chromosome pairing in wheat and *E. elongata* hybrid progenies.

## Introduction

Modern cultivation strategies have diminished the genetic base of common wheat (*Triticum aestivum*). A number of wild relatives and related species were popularly used to increase the genetic diversity available to wheat breeders. *Elytrigia elongata* (Host) Nevisk. [Syn. *Thinopyrum ponticum* (Podp.) Barkworth] (2*n* = 10x = 70) was initially hybridized with wheat approximately 70 years ago because of its resistance to several wheat diseases, as well as its stress tolerance and high crossing ability with various *Triticum* species (Sepsi, 2010; Hu et al., 2011; Fu et al., 2012; Ayala-Navarrete et al., 2013; He et al., 2013; Zheng et al., 2014; Li et al., 2016). Many desirable genes, such as Sr25, Sr43, Lr19, Cmc2, and Pm51, have been characterized and transferred from this wild grass species into wheat. These translocations have supported the development of several wheat germplasms that are used in wheat improvement programs throughout the world (Li and Wang, 2009; Niu et al., 2014; Zhan et al., 2014). The genomic composition of the decaploid species *E. elongata* has been a subject of interest for quite some time and is designated JJJJJJJ^s^J^s^J^s^J^s^ (Chen et al., 1998) or StStStStE^e^E^e^E^b^E^b^E^x^E^x^ (Zhang et al., 1996). There is some evidence that the St chromosomes in *E. elongata* are closely related to those of *Pseudoroegneria strigosa* and that the J/E^b^ and J^s^/E^e^ genomes are closely related to the *Thinopyrum bessarabicum* and/or *Thinopyrum elongatum* genomes (Chen et al., 2001). However, the genomic composition of *E. elongata* has not yet been clarified.

Chromosome engineering is the procedure of altering ploidy, chromosome structure, and/or chromosome number of an organism intended for genetic improvement. This technology has been used to incorporate favorable genes from wild species into the wheat genome for germplasm and variety development. These favorable genes can be introduced into wheat from wild species through chromosome addition, substitution, and translocation. Alien chromosome addition and substitution, which introduce one or more entire foreign chromosomes into the wheat genome, usually include desirable genes, as well as undesirable genes. There is a general demand to quickly utilize those lines in wheat breeding. Chromosome translocation, which integrates alien chromosome segments containing the gene of interest into the wheat genome, has been the most effective approach for alien gene introgression (Guo et al., 2015; Li et al., 2016). The translocations generally result from meiotic recombination between wheat chromosomes and their homoeologous complements from wild species (Bagherikia et al., 2014; Song et al., 2016).

The corresponding chromosomes of the A, B, and D genomes are genetically closely related. However, the pairing propinquity between genetically analogous chromosomes of these genomes is suppressed, largely by the activity of the *Ph1* gene in the long arm of chromosome 5B (Sears, 1976). *Ph1* represses homoeologous pairing so that only homologous partners can pair. So far, allelic variation inducing different levels of homoeologous pairing in wheat or in wheat hybrids has not been found in *Ph1*. Such variation can best be discovered in intergeneric hybrids where homologs are not present and homoeologous pairing is normally very low so that any change in the level of pairing can be demonstrably detected. While in several intergeneric hybrids, the action of *Ph1* is counterbalanced by pairing promoters of the alien species, and in most intergeneric wheat hybrids there is either little or no effect of the alien genome on homoeologous pairing (Qi et al., 2007).

Metaphase I (MI) pairing reflects cross-formation that might be associated with recombination. Metamorphic chromosomal pairing from meiosis between interspecific or intraspecific hybrids is an efficient method for estimating interphase gene transfer and revealing phylogenetic relationships among these species (Bao et al., 2014; Su et al., 2016). Cytogenetic studies on intergeneric hybrids between *Elytrigia* species have shown close relationships between J/E^b^, J^s^/E^e^, and St chromosomes (Chen et al., 2001; Liu et al., 2007). Although some information on chromosome pairing in *Elytrigia* and wheat hybrids is available (Roundy, 1985; Cai and Jones, 1997), little is known about the pairing frequency between *E. elongata* and wheat chromosomes because of the complexity of wheat-*E. elongata* chromosome pairings and the difficulty of distinguishing chromosomes in hybrids using conventional chromosome techniques.

In this study, hybrid progeny involving F_1_, F_2_, BC_1_F_1_, BC_1_F_2_, and BC_2_F_1_ were created by hybridizing *T. aestivum* with *E. elongata* to transfer desirable traits from *E. elongata* into wheat. The objective of this work was to characterize the meiotic behavior and genomic composition of the progeny from wheat-*E. elongata* hybrids using cytogenetic analysis and genomic *in situ* hybridization (GISH) technology.

## Materials and methods

### Plant material

**Figure 1:**
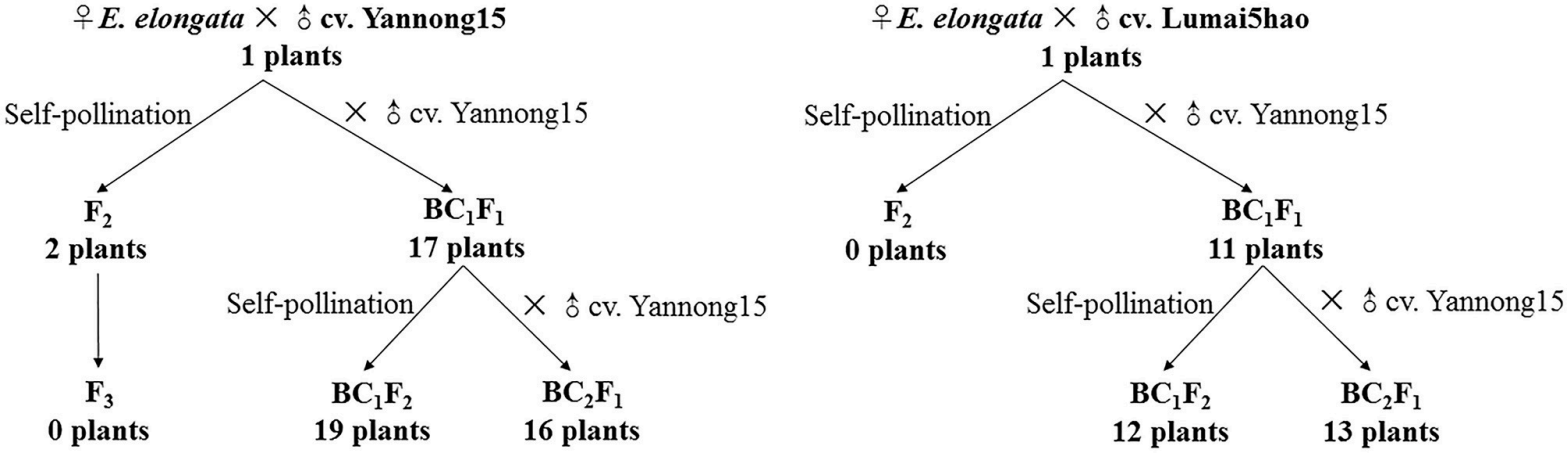
Scheme of crosses between *E. elongata* × *T. aestivum*.

*E. elongata* was provided by Prof. Zhensheng Li, formerly of the Northwest Institute of Botany, Chinese Academy of Sciences, Yangling, China. The *E. elongata* × *T. aestivum* (cv. Yannong15) and *E. elongata* × *T. aestivum* (cv. Lumai5hao) were obtained from Prof. Honggang Wang (College of Agronomy, Shandong Agricultural University, Taian, China). All plant materials were maintained through selfing at the Tai'an Subcenter of the National Wheat Improvement Center, Shandong, China. The crosses and results of offspring production are described in Figure 1.

### Meiotic preparations

When the plants reached the flag leaf stage, spikes were sampled, stages of meiosis were determined in acetocarmine squashes of 1 of 3 anthers per flower. If appropriate stages were present, the remaining 2 anthers were fixed in ethanol-acetic acid (3:1) for 24 h and stored at 4°C in 70% alcohol until use. Preparations were made from pollen mother cells (PMCs) by squashing pieces of anthers in 45% acetic acid. Slide preparations were examined using phase-contrast microscopy and then placed on dry ice to remove the cover glass. The images were captured with an Olympus BX-60.

### Gish techniques

*Elytrigia elongata* DNA was labeled with fluorescein-12-dUTP by nick translation to be used as a probe. Sheared genomic DNA from Yannong15 (AABBDD, 2*n* = 42) was used as blocking DNA. Detailed procedures of the hybridization mixture were performed as previously described (Kato et al., 2004). The slides were counterstained with propidium iodide (PI, 0.25 mg/mL) in Vectashield mounting medium (Vector Laboratories, USA).

### Statistical analyses

The data concerning the number of univalents, bivalents, trivalents, quadrivalents, pentavalents, and hexavalents for all PMCs of BC_1_F_1_, BC_1_F_2_, and BC_2_F_1_ hybrids studied were considered binomial responses, with the appropriate totals, obtained in a one-way classification. They were analyzed by the generalized linear model with logit link function to estimate mean values for plants and to test the significance of differences between plants. The calculation of mean values, standard deviations and coefficient of variation were analyzed by Excel 2013 with the statistics function. ANOVA analysis was carried out using Excel 2013, and the statistical significance (P) is shown in the Tables S1–S3.

## Results

### Chromosome pairing in F_1_ hybrids

**Figure 2:**
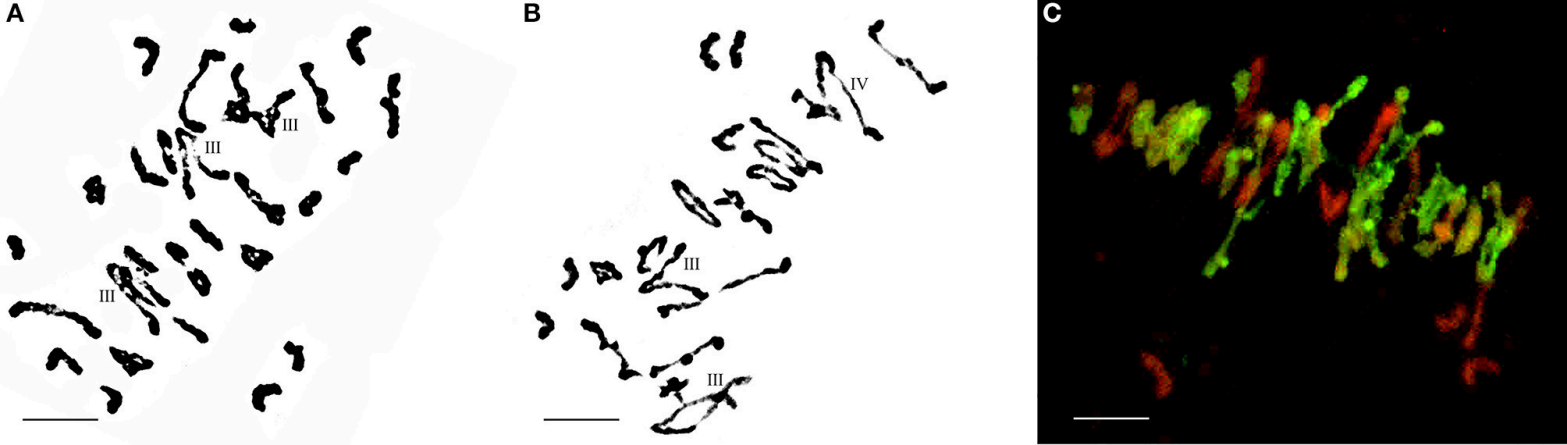
Chromosome configuration of PMC MI in wheat-*E. elongata* F_1_ hybrids. **(A)** Chromosome configurations of F_1_-1: 2*n* = 7I+20II+3III; **(B)** Chromosome configurations of F_1_-2: 2*n* = 6I+20II+2III+1IV; **(C)** *E. elongata* chromosome configurations of F_1_-1: 2*n* = 3I+16II. Wheat chromosomes were detected in red and *E. elongata* chromosomes or chromosome segments were visualized in green. Bar = 10 μm

The F_1_ hybrids from the *E. elongata* × *T. aestivum* cross exhibited a low setting percentage and were morphologically different from the 2 parents, except for a similar perennial of *E. elongata*. All plants had 56 somatic chromosomes with 35 chromosomes from *E. elongata*. Meiotic association was determined in 29 PMCs at the MI stage from *E. elongata* × *T. aestivum* cv. Yannong15 (F_1_-1) and 37 PMCs at the MI stage from *E. elongata* × *T. aestivum* cv. Lumai5hao (F_1_-2) (Table 1), and the average chromosome configurations were 14.96I+17.8II+0.69III+0.63IV+0.17V (F_1_-1, Figure 2A) and 18.02I+16.61II+0.61III+0.57IV+0.13V (F_1_-2, Figure 2B), respectively. Chromosome pairing configurations in the hybrid PMCs were very complex, and a high frequency of univalent and a variety of trivalent and tetravalent configurations were observed.

**Table 1:** Chromosome configuration of PMC MI in wheat-*E. elongata* F_1_ hybrids.

**Lines**	**Cells No. of cytogenetic analysis**	**Chromosome No**.	**Average chromosome configurations**					**Average Chromosome configurations of** ***E. elongata***					**Average Chromosome configurations of wheat*****-E. elongata***				***E. elongata* chromosome No**.	**Cells No. of GISH**	**Seed setting rate**
			**I**	**II**	**III**	**IV**	**V**	**I**	**II**	**III**	**VI**	**V**	**II**	**III**	**VI**	**V**			
F_1_-1	29	56	14.96	17.8	0.69	0.63	0.17	11.03	9.81	0.37	0.61	0.16	0.41	0.09	0.03	0.02	35	31	0.17%
			(6–19)	(15–23)	(0–5)	(0–2)	(0–2)	(3–13)	(7–17)	(0–3)	(0–1)	(0–1)	(0–2)	(0–1)^a^	(0–1)^b^	(0–1)^c^			
F_1_-2	37	56	18.02	16.61	0.61	0.57	0.13	14.45	8.4	0.33	0.54	0.14	0.38	0.08	0.02		35	24	0
			(7–21)	(14–22)	(0–4)	(0–2)	(0–2)	(5–16)	(6–16)	(0–2)	(0–1)	(0–1)	(0–2)	(0–1)^a^	(0–1)^b^				
mean value			16.49	17.21	0.65	0.60	0.15	12.74	9.11	0.35	0.58	0.15	0.40	0.09	0.025	0.01			

a *W/W/E*.

b *W/W/E/E*.

c *W/W/W/E/E*.

GISH was performed to detect *E. elongata* chromosomes in F_1_-1 and F_1_-2 (Figure 2C) using total genomic DNA from *E. elongata* as a probe and ABD-genomic DNA from Yannong15 wheat as a blocker. The mean *E. elongata* chromosome configurations determined after GISH analysis were 11.03I+9.81II+0.37III+0.61IV+0.16V and 14.45I+8.4II+0.33III+0.54IV+0.12V, respectively (Table 1). The chromosome configurations of wheat-*E. elongata* in the hybrid included bivalents, one type of trivalent (W/W/E), one chain quadrivalent (W/W/E/E), and one chain pentavalent (W/W/W/E/E) (Table 1).

### Chromosome pairing in F_2_ progeny

**Figure 3:**
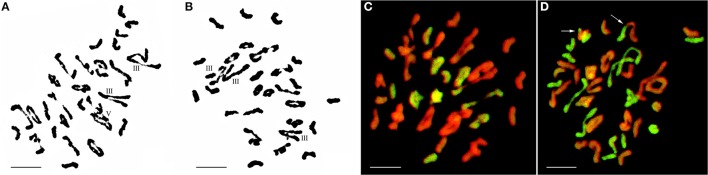
Chromosome configuration in PMC MI for wheat-*E. elongata* F_2_ hybrids. **(A)** Chromosome configuration of F_2_-1: 2*n* = 10I+14II+2III+1V; **(B)** Chromosome configuration of F_2_-2: 2*n* = 7I+18II+3III; **(C)** *E. elongata* chromosome configuration of F_2_-1: 2*n* = 5I+6II; **(D)** *E. elongata* chromosome configuration of F_2_-2: 2*n* = 10I+5II. Wheat chromosomes were detected in red and *E. elongata* chromosomes or chromosome segments were visualized in green. The arrows indicate pairing between wheat and *E. elongata* chromosomes. Bar = 10 μm.

Although five of the F_1_-1 selfed F_2_ seeds were obtained, wherein two survived, the F_1_-2 and these two F_2_ plants were self-sterile. These F_2_ plants (F_2_-1 and F_2_-2) were identified by cytogenetic analysis and GISH (Table 2). The F_2_-1 plant had 49 chromosomes, 18 of which were from *E. elongata*, and the F_2_-2 plant had 52 chromosomes, 20 of which were from *E. elongata*. The average chromosome configurations were 11.22I+15.32II+0.93III+0.41IV+0.29V+0.21VI (F_2_-1, Figure 3A) and 11.92I+16.5II+0.79III+0.37IV+0.37V+0.23VI (F_2_-2, Figure 3B), respectively. GISH analysis showed that the average *E. elongata* chromosome configurations were 5.39I+5.68II+0.27III+0.11IV (F_2_-1, Figure 3C) and 8.87I+5.337II+0.152III (F_2_-2, Figure 3D), respectively. The chromosome configurations of wheat-*E. elongata* in the hybrid included bivalents, one kind of trivalent (W/W/E), and one chain quadrivalent (W/W/W/E) (Table 1). In addition, a translocation or interspecific chromosome pairing between wheat and *E. elongata* chromosomes was also detected in some of these plants (Figure 3D, arrows).

**Table 2:** Chromosome configuration of PMC MI in wheat-*E. elongata* F_2_ hybrids.

**Lines**	**Cells No. of cytogenetic analysis**	**Chromosome No**.	**Average chromosome configurations**						**Average Chromosome configurations of** ***E. elongata***				**Average Chromosome configurations of wheat*****-E. elongata***			***E. elongata* chromosome No**.	**Cells No. of GISH**
			**I**	**II**	**III**	**IV**	**V**	**VI**	**I**	**II**	**III**	**IV**	**II**	**III**	**IV**		
F_2_-1	47	49	11.22	15.32	0.93	0.41	0.29	0.21	5.39	5.68	0.27	0.11	0.21	0.03	0.03	18	33
			(7–13)	(11–17)	(0–4)	(0–2)	(0–2)	(0–1)	(3–8)	(3–7)	(0–2)	(0–1)	(0–2)	(0–1)^a^	(0–1)^b^		
F_2_-2	52	52	11.92	16.5	0.79	0.37	0.37	0.23	8.87	5.337	0.152		0.13	0.03	0.03	20	36
			(6–14)	(14–21)	(0–4)	(0–3)	(0–2)	(0–1)	(6–11)	(3–8)	(0–2)		(0–2)	(0–1)^a^	(0–1)^b^		
mean value			11.57	15.91	0.86	0.39	0.33	0.22	7.13	5.509	0.211	0.11	0.17	0.03	0.03		

a *W/W/E*.

b *W/W/W/E*.

### Chromosome pairing and separation trend in hybrid derivatives

**Figure 4:**
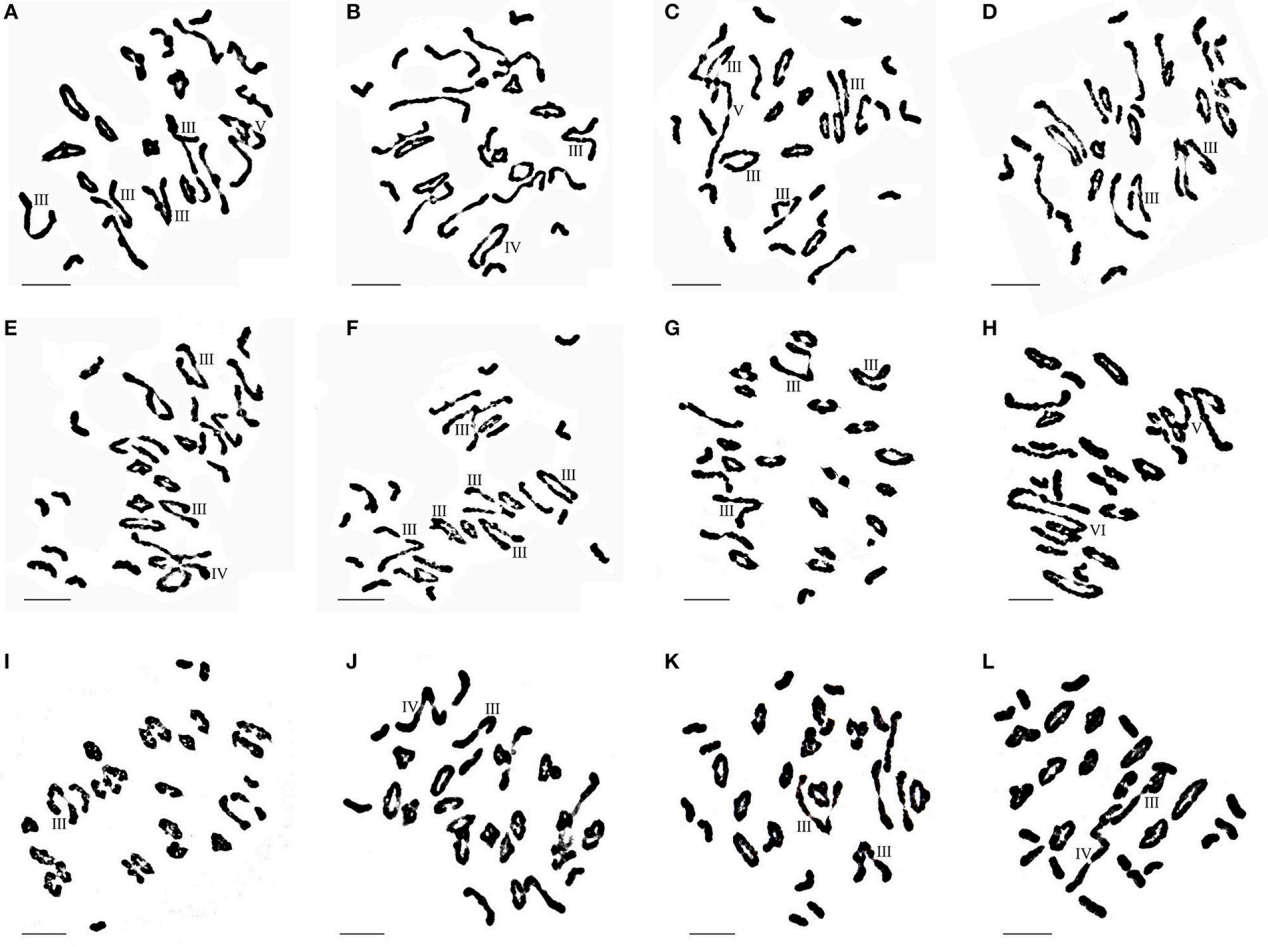
Chromosome configuration of wheat- *E. elongata* BC_1_F_1_, BC_1_F_2_ and BC_2_F_1_ hybrids. **(A–F)** Chromosome configurations in BC_1_F_1_ **(A**) 2*n* = 3I+15II+4III+1IV; **(B)** 2*n* = 6I+17II+1III+1IV; **(C)** 2*n* = 12I+10II+4III+1V; **(D)** 2*n* = 8I+16II+2III; **(E)** 2*n* = 11I+12II+2III+1IV; **(F)** 2n=10I+9II+6III; **(G–J)** Chromosome configurations in BC_1_F_2_ **(G)** 2*n* = 3I+19II+3III; **(H)** 2*n* = 5I+14II+1V+1VI; **(I)** 2*n* = 7I+21II+1III; **(J)** 2*n* = 3I+20II+1III+1IV; **(K–L)** Chromosome configurations in BC_2_F_1_ **(K)** 2*n* = 10I+13II+2III; **(L)** 2*n* = 15I+11II+1III+1IV. Bar = 10 μm.

**Figure 5:**
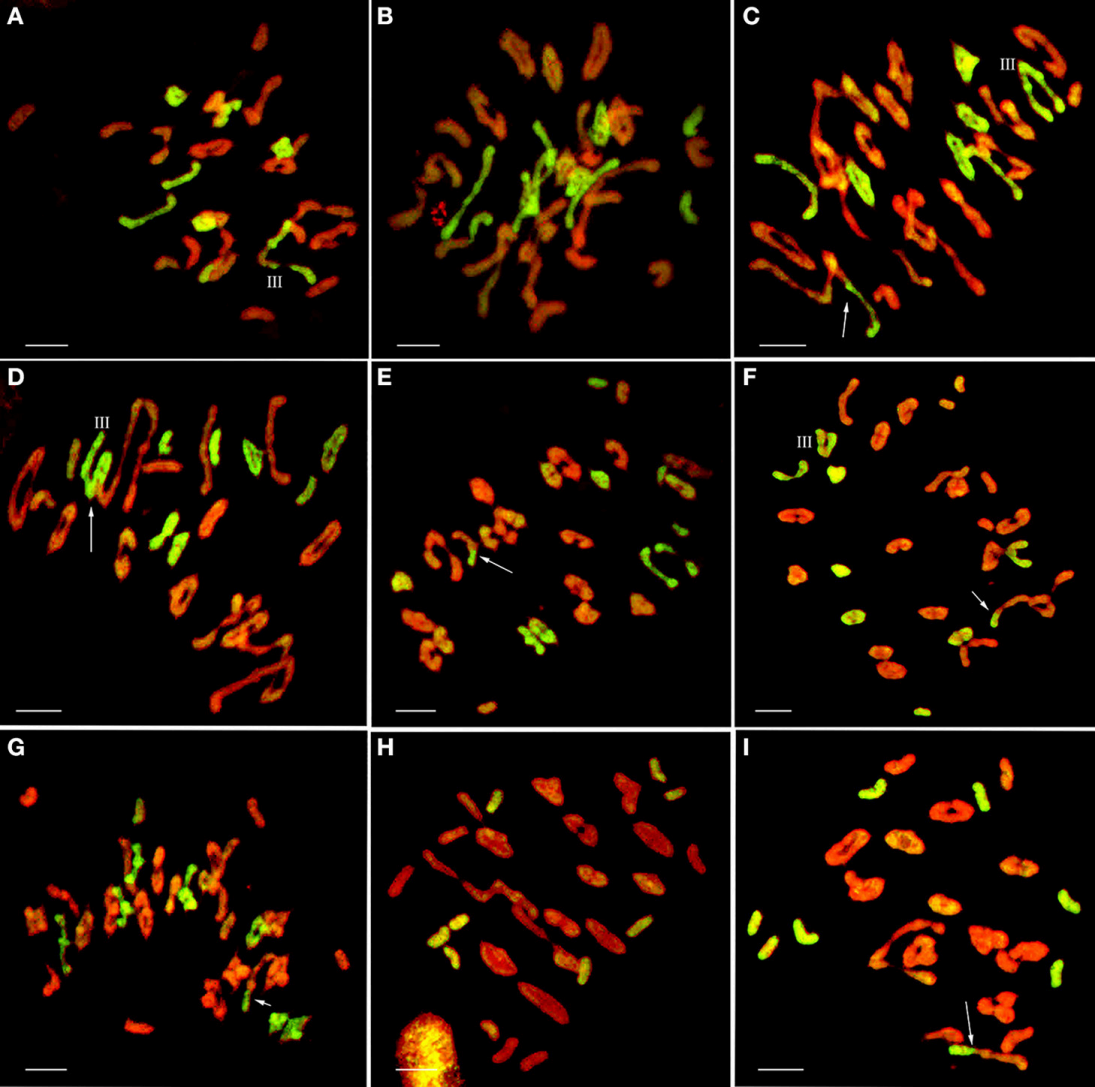
GISH patterns of PMC MI in wheat-*E. elongata* BC_1_F_1_, BC_1_F_2_, and BC_2_F_1_ hybrids**. (A,B)** *E. elongata* chromosome configurations in BC_1_F_1_ **(A)** 2*n* = 1I+6II+1III; **(B)** 2*n* = 3I+7II; **(C–G)** *E. elongata* chromosome configurations in BC_1_F_2_ **(C)** 2*n* = 1I+6II+1III; **(D)** 2*n* = 3I+4II+1III; **(E)** 2*n* = 5I+6II; **(F)** 2*n* = 2I+6II+1III; **(G)** 2*n* = 3I+7II; **(H–I)** *E. elongata* chromosome configurations in BC_2_F_1_ **(H)** 2*n* = 8I; **(I)** 2*n* = 8I. Wheat chromosomes were detected in red and *E. elongata* chromosomes or chromosome segments were visualized in green. The arrows indicate pairing between wheat and *E. elongata* chromosomes. Bar = 10 μm.

Seventeen plants were produced from F_1_-1 hybrids with *T. aestivum* cv. Yannong15, and 11 plants were produced from F_1_-2 hybrids with *T. aestivum* cv. Yannong15. The PMCs from these 28 BC_1_F_1_ hybrid plants were analyzed with cytogenetic and GISH techniques (Table S1). The mean chromosome number of the BC_1_F_1_ progeny was 2*n* = 48.25. Most lines (18 plants) had 2*n* = 47–49; the distribution range was 44–52 (Table 3). The combinations of average chromosome configurations included 6.17–11.92 univalents, 15.07–18.6 bivalents, 0.31–1.52 trivalents, 0.1–0.79 tetravalents, 0–0.41 pentavalents and 0–0.23 hexavalents (Table S1, Figures 4A–F). GISH analysis revealed that 10–20 *E. elongata* chromosomes were detected in BC_1_F_1_ progeny (Table 3); the distribution range of average *E. elongata* chromosome configurations was 1.96–8.87 univalents, 2.62–6.41 bivalents, 0.12–1.04 trivalents, and 0.12–1.04 tetravalents (Table S1, Figures 5A,B). The average pairing configuration for wheat-*E. elongata* chromosomes included 0.15–0.32 bivalents, 0.02–0.06 trivalents, 0–0.03 tetravalents, and 0–0.04 pentavalents (Table S1).

**Table 3:** Chromosome segregation trends in wheat-*E. elongata* BC_1_F_1_, BC_1_F_2_, and BC_2_F_1_ hybrids.

**Lines**	**Chromosome No**.														**Plants No**.	***E. elongata*** **chromosome No**.													
	**42**	**43**	**44**	**45**	**46**	**47**	**48**	**49**	**50**	**51**	**52**	**53**	**54**	**55**		**6**	**7**	**8**	**9**	**10**	**11**	**13**	**14**	**15**	**16**	**17**	**18**	**20**	**21**
BC_1_F_1_	–	–	1	–	3	5	9	4	3	–	3	–	–	–	28	–	–	–	–	1	1	2	3	7	9	2	2	1	–
BC_1_F_2_	1	1	1	1	1	2	2	2	2	5	4	4	4	1	31	–	1	–	1	–	–	1	9	4	7	4	3	–	1
BC_2_F_1_	5	3	5	5	3	3	2	2	–	–	–	–	–	–	29	3	5	9	7	3	2	–	–	–	–	–	–	–	–

Thirty-one BC_1_F_2_ plants were randomly selected from BC_1_F_1_ self-fertilization progeny for further cytogenetic analysis. The mean chromosome number of the progenies was 2*n* = 50.13; the distribution range was 42–55 (Table 3). The distribution range of average chromosome configuration at meiotic metaphase I in BC_1_F_2_ PMCs included 2.51–16.01 univalents, 11.01–24.25 bivalents, 0.17–2.67 trivalents, 0–1.37 quadrivalents, 0–1.17 pentavalents and 0–0.83 hexavalents (Table S2, Figures 4G–J). GISH analysis during meiosis revealed 7-21 chromosomes with hybridization signals in these 31 plants (Table 3). The average pairing configuration of *E. elongata* chromosomes included 0.34–6.69 univalents, 0.5–7.94 bivalents, 0–1.14 trivalents and 0–0.54 tetravalents (Table S2, Figures 5C–G). The distribution range of average wheat-*E. elongata* chromosome configurations was 0.15–0.3 bivalents, 0.02–0.07 trivalents, 0–0.04 tetravalents, and 0–0.05 pentavalents (Table S2).

Twenty-nine BC_2_F_1_ plants produced from BC_1_F_1_ hybrids with *T. aestivum* cv. Yannong15 were analyzed by cytogenetic techniques and GISH. Overall, 42–50 total chromosomes and 6–11 *E. elongata* chromosomes were detected in these plants (Table 3). The distribution range of average chromosome configurations included 8.21–11.77 univalents, 12.68–17.34 bivalents, 0–1.77 trivalents, 0–1.38 tetravalents, 0–0.31 pentavalents and 0–0.1 hexavalents (Table S3, Figures 4K,L). GISH analysis revealed that the average pairing configuration for *E. elongata* chromosomes included 0.33–0.67 univalents, 4.21–9.64 bivalents, and 0.15–2.39 trivalents (Table S3, Figure 5H,I). The average pairing configuration of wheat-*E. elongata* chromosomes included 0.17–0.39 bivalents, 0.03–0.14 trivalents, 0–0.04 tetravalents, and 0–0.04 pentavalents (Table S3).

The separation trend is the chromosome variation amplitude of total chromosome number and *E. elongata* chromosome number of BC_1_F_2_ and BC_2_F_1_ compared with BC_1_F_1_. Obviously, the number of bivalents, trivalents and tetravalents among BC_1_F_1_, BC_1_F_2_, and BC_2_F_1_ plants were different. The numbers of chromosomes increased after selfing according to the result, and the pairing chromosome number also increased after selfing and backcrossing. Additionally, exogenous chromosomes decreased after backcrossing. These results were consistent with the theoretical hypothesis.

## Discussion

*E. elongata* is an influential perennial *Triticeae* species with a considerable number of traits with the potential to improve wheat. Several studies have reported wide hybridization between *E. elongata* and other species of Triticeae (Fu et al., 2012; Ayala-Navarrete et al., 2013; Guo et al., 2015). A higher seed set was usually obtained when *T. aestivum* was used as the female parent, whereas hybrid seed development was usually less successful. In wide hybridization between wheat and *E. elongata*, a 15.9% (0–76.9%) average seed setting rate in the dozens of combinations showed a very low crossability (Group of Eemote and Northwestern Institute, 1977). It is difficult to obtain offspring from the wheat and *E. elongata* hybrid; over the years, we have only obtained two perennial F_1_ plants. Early studies in our laboratory found that, in distant hybridization, when *T. aestivum* cv. Yannong15 was a parent, the seed setting rate and seed survival rate of the offspring were the highest. Therefore, in order to obtain more seeds, we use *T. aestivum* cv. Yannong15 as a backcross parent. In this study, we harvested only five seeds from *E. elongata* × *T. aestivum* cv. Yannong15 offspring, and only two survived. This may be due to the genome ploidy gap between wheat and *E. elongata*, although the genetic relationship between them is very close, and may also be caused by the difference between common wheat varieties.

In recent decades, several wheat-*E. elongata* amphiploid, addition, substitution, and translocation lines have been developed in various laboratories throughout the world and are promising sources of multiple disease resistance (Fu et al., 2012; Zheng et al., 2014; Li et al., 2016). However, few studies have focused on the transmission characteristics of *E. elongata* chromosomes in the *T. aestivum* background. GISH has proved to be a useful technique to genetically differentiate closely related genomes, to distinguish alien chromosomes from wheat chromosomes, and to identify wheat-alien translocated chromosomes in a wheat background (Jiang and Gill, 2006; Scoles et al., 2010; Guo et al., 2015). In this study, GISH using *E. elongata* DNA as a probe was a powerful tool to differentiate chromosomes from *T. aestivum* and *E. elongata* hybrid progeny in PMCs at the MI stage. This differentiation allowed the precise analysis of the chromosome composition and the relationships between *E. elongata* and wheat chromosomes in a wheat genetic background. Using this approach, the genomic composition of the wheat*-E. elongata* BC_1_F_1_, BC_1_F_2_, and BC_2_F_1_ hybrid progenies was clearly identified in the MI stage and was shown to contain 10–20, 7–21, and 6–11 *E. elongata* chromosomes, respectively. In the backcross generation, the number of *E. elongata* chromosomes decreased rapidly; the distribution of *E. elongata* chromosomes was more extensive in self-progeny. This observation indicated that backcrossing will promote cytological stability and that inbreeding will increase variability.

The genomic composition of *E. elongata* has been reported to be decaploid, with the genomic designation JJJJJJJ^s^J^s^J^s^J^s^ (Chen et al., 1998) or StStStStE^e^E^e^E^b^E^b^E^x^E^x^ (Zhang et al., 1996). The F_1_ hybrids were expected to have the genomic constitution of ABDJJJJ^s^J^s^ or ABDStStE^e^E^b^E^x^ (2*n* = 56 chromosomes), and the theoretical *E. elongata* chromosome configuration of these F_1_ should be 7II+7III (JJJJ^s^J^s^) or 21I+7II (StStE^e^E^b^E^x^). In this study, the average *E. elongata* chromosome configurations of F_1_ hybrids after GISH analysis were 11.03I+9.81II+0.37III+0.61IV+0.16V and 14.45I+8.4II+0.33III+0.54IV+0.12V. The earlier conclusion that the St and J/E^b^ (including J/E^b^ and J^s^/E^e^) genomes are very closely related was drawn from molecular and cytogenetic studies (Liu et al., 2007; Mahelka et al., 2013; Kantarski et al., 2017; Linc et al., 2017). In meiotic metaphase I, these closely related chromosomes may be associated with allosyndetic pairing, thereby reducing the number of univalents and increasing the number of bivalents and multivalents. Thus, in the actual statistical chromosome configuration, the univalents will be less than the theoretical value, while the bivalents and multivalents will be greater than the theoretical value. The average *E. elongata* chromosome configuration of these two F_1_ lines accorded with the theoretical chromosome configuration of 21I+7II. Therefore, the genomic composition of *E. elongata* should be StStStStE^e^E^e^E^b^E^b^E^x^E^x^.

The strict pairing of homologous chromosomes in hexaploid wheat reflects a delicate balance between genes that inhibit homologous pairing, such as *Ph1* and *Ph2*, and genes that promote pairing, such as those located on homologous groups 2, 3, and 5 (Naranjo and Benavente, 2015). A similar theory was suggested for *Elytrigia* species. Dvorák (1987) proposed that the chromosome arms 3ES, 3EL, 4ES, and 5Ep and chromosome 6E of *T. elongatum* had genes that induce homoeologous chromosome pairing. Charpentier et al. (1988) further demonstrated that the role of 5E in the wheat and *Agropyron elongatum* hybrid was similar to the deletion of the *Ph1* gene. Later, Zhang et al. (1995) implied that two basic chromosomes in *E. elongata* encode genes that promote homoeologous chromosome pairing and might have additive effects. Although more recent studies observed similar inferences, there is no direct evidence to confirm these hypotheses. In the present study, pairing between wheat and *E. elongata* was detected in each of the wheat*-E. elongata* hybrid progenies, albeit rarely. This result suggests a close genetic relationship between wheat and *E. elongata* chromosomes. Similar results were detected on meiotic chromosomes at MI in trigeneric hybrids produced from a heterozygous Langdon *Ph* mutant (*Ph1ph1b*) or Langdon 5D (5B) disomic substitution line (without *Ph1*) hybridization with the JJEE amphidiploids using multicolor fluorescent GISH by Jauhar et al. (2004) and (Jauhar and Peterson (2006)). The pairing between wheat and *E. elongata* chromosomes can be used as direct evidence that genes promoting homoeologous chromosome pairing or *Ph* suppressor genes exist in *E. elongata*. Although it is worthwhile for *E. elongata* chromosomes to promote homoeologous pairing or inhibit *Ph* gene effects, the use of these genotypes might promote the homoeologous pairing of *E. elongata* and wheat chromosomes and facilitate alien gene transfer into the wheat genome.

Common wheat is a major, global cereal crop that accounts for approximately 20% of the calories consumed by humans (Brenchley et al., 2012). However, effective wheat breeding has been hindered by a narrow genetic base (Friebe et al., 1996). Genes from wild relatives have been exploited to confer desirable agronomic traits to wheat, as illustrated by the application of many wheat-alien translocation lines (Lukaszewski, 2001). For example, *Lr26*/*Sr31*/*Yr9*/*Pm8* have endowed the translocation line T1RS·1BL with improved environmental adaption and enhanced kernel numbers (Friebe et al., 1996). Both *T. aestivum*-*Thinopyrum bessarabicum* T2JS-2BS·2BL and *T. aestivum*-*Dasypyrum villosum* T2VS·2DL translocation lines have been reported with elevated grain numbers per spike (Qi et al., 2010; Zhang et al., 2015). However, the formation of these translocation lines is rarely reported. GISH patterns of meiotic chromosomes at MI in these hybrids of wheat with *E. elongata* indicated that chromosome pairing in these hybrids mainly occurred among wheat chromosomes and among *E. elongata* chromosomes and that allosyndetic pairing between wheat and *E. elongata* chromosomes was very rare (Table 1). The much higher frequencies of autosyndetic pairing than allosyndetic pairing in these hybrids of wheat with *E. elongata* demonstrated that the relationships among *T. aestivum* genomes and among *E. elongata* genomes are much closer than the relationship between *T. aestivum* and *E. elongata* genomes. Meanwhile, these allosyndetic pairings promote the recombination between homologous chromosomes, enrich the genetic diversity of distant hybrid progeny, and improve the frequency of the offspring to obtain a translocation line, which will benefit from the selection of excellent genetic resources, and thus applied to wheat breeding. Our results demonstrate that GISH using *E. elongata* genomic DNA as a probe provided a reliable approach to discriminate the identity of chromosomes involved in pairing. This observation might significantly improve our understanding of the genomic relationships within *Triticeae*. Knowledge of the relationships between wheat and grass genomes also improves our understanding of characteristic inheritance to generate efficient strategies for transferring target gene(s) from *E. elongata* to wheat. With the advancement and development of technology, multicolor GISH (mcGISH) has been widely used in academic research to simultaneously visualize two or more genomes in a polyploid species (Zheng et al., 2014; Guo et al., 2015). Although there are few reports analyzing chromosome pairing behavior using mcGISH, our future research will focus on these types of analyses. This approach might extend the analysis of chromosomes, genomes and phylogenies, especially for the analysis of complex polyploids and their hybrids in wheat.

## Author contributions

FH, PX, and YB performed the experiments, analyzed the data and wrote the manuscript. MR, SL, YW, XL, and HW designed the study and discussed the manuscript.

### Conflict of interest statement

The authors declare that the research was conducted in the absence of any commercial or financial relationships that could be construed as a potential conflict of interest.
