# Supplementary material for: Chromosome Pairing in Hybrid Progeny between Triticum aestivum and Elytrigia elongata
Source: Front Plant Sci. 2017 Dec 19;8:2161. doi: 10.3389/fpls.2017.02161 (PMC5742266; doi:10.3389/fpls.2017.02161)
Supplement: Supplementary file 1 [file Table1.DOCX]

**Chromosome pairing in hybrid progeny between *Triticum aestivum* and *Elytrigia elongata***

**Fang He^1^, Piyi Xing^2^, Yinguang Bao^2^, Mingjian Ren^1^, Shubing Liu^2^, Yuhai Wang^3^, Xingfeng Li^2^*, Honggang Wang^2^***

1 Guizhou Subcenter of National Wheat Improvement Center, College of Agronomy, Guizhou University, Guiyang 550025, People’s Republic of China

2 State Key Laboratory of Crop Biology, Shandong Key Laboratory of Crop Biology, College of Agronomy, Shandong Agricultural University, Taian 271018, People’s Republic of China

3 Zaozhuang University, Zaozhuang 277160, People’s Republic of China

* Corresponding author. *E-mail address*: lixf@sdau.edu.cn and [hgwang@sdau.edu.cn](mailto:hgwang@sdau.edu.cn)

**Table S1 Chromosome configuration of PMC MI in wheat-*E. elongata* BC_1_F_1_ hybrids**

| Lines | Observed Cells No. | Average chromosome configurations | | | | | | Chromosome No. | Observed Cells No. | Average Chromosome configurations of *E. elongata* | | | | *E. elongata* chromosome No. | Average Chromosome configurations of wheat-*E. elongata* | | | |
| --- | --- | --- | --- | --- | --- | --- | --- | --- | --- | --- | --- | --- | --- | --- | --- | --- | --- | --- |
|  |  | Ⅰ | Ⅱ | Ⅲ | Ⅳ | Ⅴ | Ⅵ |  |  | Ⅰ | Ⅱ | Ⅲ | Ⅳ |  | Ⅱ | Ⅲ | Ⅳ | V |
| BC_1_F_1_ | 68 | 7.92 | 16.07 | 0.64 | 0.38 | 0.1 |  | 44 | 48 | 2.57 | 6.17 | 0.19 | 0.13 | 16 | 0.17 | 0.03 | 0.03 |  |
|  | 76 | 7.74 | 17.72 | 0.59 | 0.1 | 0.13 |  | 46 | 66 | 2.34 | 6.415 | 0.17 | 0.08 | 16 | 0.18 | 0.03 |  |  |
|  | 56 | 6.91 | 17.771 | 0.44 | 0.31 | 0.11 | 0.073 | 46 | 49 | 2.91 | 5.575 | 0.22 | 0.07 | 15 | 0.17 | 0.04 | 0.02 | 0.02 |
|  | 87 | 7.57 | 17.475 | 0.63 | 0.21 | 0.15 |  | 46 | 69 | 1.96 | 6.06 | 0.64 |  | 16 | 0.17 | 0.02 |  |  |
|  | 75 | 9.2 | 16.155 | 1.43 | 0.3 |  |  | 47 | 68 | 3.91 | 5.865 | 0.12 |  | 16 | 0.18 | 0.05 | 0.02 |  |
|  | 65 | 9.76 | 16.33 | 0.86 | 0.5 |  |  | 47 | 55 | 4.37 | 5.885 | 0.62 |  | 18 | 0.18 | 0.03 |  |  |
|  | 68 | 9.11 | 15.07 | 1.52 | 0.33 | 0.17 | 0.17 | 47 | 51 | 2.07 | 3.905 | 1.04 |  | 13 | 0.17 | 0.03 | 0.02 | 0.04 |
|  | 76 | 9.76 | 16.39 | 0.86 | 0.47 |  |  | 47 | 56 | 3.11 | 4.075 | 0.94 | 0.23 | 15 | 0.2 | 0.03 |  |  |
|  | 75 | 8.93 | 15.59 | 1.31 | 0.39 | 0.28 |  | 47 | 45 | 1.98 | 4.995 | 1.01 |  | 15 | 0.18 | 0.02 |  |  |
|  | 58 | 6.82 | 18.6 | 0.62 | 0.53 |  |  | 48 | 41 | 3.65 | 2.62 | 0.37 |  | 10 | 0.18 | 0.03 | 0.02 |  |
|  | 67 | 8.61 | 18.51 | 0.59 | 0.15 |  |  | 48 | 41 | 2.48 | 3.255 | 0.67 |  | 11 | 0.18 | 0.04 | 0.02 |  |
|  | 62 | 6.29 | 18.2 | 0.81 | 0.47 | 0.2 |  | 48 | 46 | 2.63 | 4.4 | 0.27 | 0.44 | 14 | 0.2 | 0.03 |  |  |
|  | 61 | 6.17 | 17.18 | 1.1 | 0.58 | 0.37 |  | 48 | 39 | 2.17 | 5.03 | 0.67 | 0.19 | 15 | 0.31 | 0.03 |  |  |
|  | 45 | 9.3 | 18.16 | 0.31 | 0.2 | 0.13 |  | 48 | 41 | 4.17 | 5.505 | 0.18 | 0.07 | 16 | 0.18 | 0.04 | 0.03 | 0.03 |
|  | 56 | 7.94 | 17.33 | 0.73 | 0.41 | 0.23 | 0.07 | 48 | 51 | 3.25 | 6.2 | 0.45 |  | 17 | 0.17 | 0.02 |  |  |
|  | 45 | 7.33 | 17.18 | 1.11 | 0.57 | 0.14 |  | 48 | 39 | 2.95 | 4.4 | 0.75 |  | 14 | 0.22 | 0.05 | 0.02 | 0.02 |
|  | 51 | 9.08 | 16.27 | 0.83 | 0.61 | 0.29 |  | 48 | 37 | 2.41 | 5.48 | 0.37 | 0.13 | 15 | 0.31 | 0.05 |  |  |
|  | 56 | 8.33 | 17.4 | 0.42 | 0.29 | 0.37 | 0.1 | 48 | 46 | 3.07 | 6.105 | 0.24 |  | 16 | 0.17 | 0.03 |  |  |
|  | 54 | 11.41 | 16.44 | 0.77 | 0.6 |  |  | 49 | 34 | 4.64 | 4.47 | 0.14 |  | 14 | 0.17 | 0.03 | 0.03 |  |
|  | 46 | 10.97 | 15.44 | 0.83 | 0.79 | 0.3 |  | 49 | 26 | 4.81 | 5.395 | 0.24 | 0.17 | 17 | 0.18 | 0.02 |  |  |
|  | 69 | 11.84 | 16.68 | 0.48 | 0.59 |  |  | 49 | 51 | 4.57 | 5.385 | 0.22 |  | 16 | 0.31 | 0.04 | 0.03 |  |
|  | 71 | 10.7 | 17.2 | 0.9 | 0.3 | 0.2 |  | 50 | 39 | 5.26 | 5.01 | 0.72 | 0.14 | 18 | 0.17 | 0.03 |  |  |
|  | 49 | 10.41 | 17.97 | 0.87 | 0.26 |  |  | 50 | 37 | 3.57 | 5.54 | 0.45 |  | 16 | 0.32 | 0.04 | 0.02 |  |
|  | 57 | 10.68 | 18.05 | 0.66 | 0.31 |  |  | 50 | 41 | 4.11 | 4 | 0.79 | 0.13 | 15 | 0.15 | 0.06 | 0.02 |  |
|  | 56 | 10.97 | 17.31 | 0.86 | 0.57 | 0.31 |  | 52 | 49 | 5.13 | 4.41 | 0.35 |  | 15 | 0.15 | 0.04 |  |  |
|  | 53 | 10.38 | 17.86 | 0.79 | 0.37 | 0.41 |  | 52 | 33 | 5.34 | 3.135 | 0.41 | 0.04 | 13 | 0.13 | 0.04 | 0.03 | 0.04 |
|  | 62 | 11.22 | 15.32 | 0.93 | 0.41 | 0.29 | 0.21 | 49 | 47 | 5.39 | 4.68 | 0.27 | 0.11 | 16 | 0.18 | 0.03 |  | 0.03 |
|  | 73 | 11.92 | 16.5 | 0.79 | 0.37 | 0.37 | 0.23 | 52 | 51 | 8.87 | 5.337 | 0.152 |  | 20 | 0.17 | 0.04 | 0.02 | 0.02 |
| mean value |  | 9.19 | 17.01 | 0.81 | 0.41 | 0.16 | 0.03 | 48.25 |  | 3.70 | 4.98 | 0.45 | 0.07 | 15.29 | 0.19 | 0.03 | 0.01 | 0.01 |
| standard deviations |  | 1.71 | 1.00 | 0.29 | 0.16 | 0.14 | 0.07 | 1.88 |  | 1.50 | 1.00 | 0.28 | 0.10 | 2.02 | 0.05 | 0.01 | 0.01 | 0.01 |
| coefficient of variation |  | 18.59 | 5.86 | 35.40 | 39.71 | 86.38 | 218.97 | 3.89 |  | 40.61 | 20.06 | 62.22 | 147.39 | 13.19 | 26.51 | 28.85 | 105.96 | 186.11 |
| P |  | <0.01 | <0.01 | <0.01 | <0.01 | <0.01 | <0.01 | <0.01 |  | <0.01 | <0.01 | <0.01 | <0.01 | <0.01 | <0.01 | <0.01 | <0.01 | <0.01 |

**Table S2 Chromosome configuration of PMC MI in wheat-*E. elongata* BC_1_F_2_ hybrids**

| Lines | Observed Cells No. | Average chromosome configurations | | | | | | Chromosome No. | Observed Cells No. | Average Chromosome configurations of *E. elongata* | | | | *E. elongata* chromosome No. | Average Chromosome configurations of wheat-*E. elongata* | | | |
| --- | --- | --- | --- | --- | --- | --- | --- | --- | --- | --- | --- | --- | --- | --- | --- | --- | --- | --- |
|  |  | Ⅰ | Ⅱ | Ⅲ | Ⅳ | Ⅴ | Ⅵ |  |  | Ⅰ | Ⅱ | Ⅲ | Ⅳ |  | Ⅱ | Ⅲ | Ⅳ | V |
| BC_1_F_2_ | 62 | 9.93 | 11.015 | 1.52 | 1.37 |  |  | 42 | 49 | 3.34 | 0.5 | 1.14 | 0.31 | 9 | 0.3 | 0.03 |  |  |
|  | 48 | 7.52 | 12.625 | 1.61 | 0.95 | 0.32 |  | 43 | 31 | 4.42 | 0.525 | 0.51 |  | 7 | 0.3 | 0.03 | 0.03 | 0.04 |
|  | 56 | 3.12 | 17.185 | 1.49 | 0.51 |  |  | 44 | 44 | 2.59 | 4.97 | 0.49 |  | 14 | 0.22 | 0.02 |  |  |
|  | 55 | 5.67 | 15 | 2.67 | 0.33 |  |  | 45 | 45 | 6.58 | 2.93 | 0.52 |  | 14 | 0.23 | 0.05 |  |  |
|  | 65 | 6.58 | 19.455 | 0.17 |  |  |  | 46 | 47 | 3.87 | 4.84 | 0.15 |  | 14 | 0.24 | 0.04 |  |  |
|  | 63 | 8.57 | 18.285 | 0.26 | 0.27 |  |  | 47 | 48 | 4.76 | 5.42 | 0.24 | 0.17 | 17 | 0.22 | 0.05 | 0.02 |  |
|  | 56 | 6.67 | 19.67 | 0.33 |  |  |  | 47 | 37 | 2.82 | 6.09 |  |  | 15 | 0.3 | 0.02 |  |  |
|  | 58 | 6.2 | 19.6 | 0.6 | 0.2 |  |  | 48 | 45 | 4.4 | 7.94 | 0.24 |  | 21 | 0.21 | 0.03 |  |  |
|  | 59 | 5.21 | 20.35 | 0.43 | 0.2 |  |  | 48 | 44 | 1.52 | 6.24 |  |  | 14 | 0.22 | 0.05 | 0.02 |  |
|  | 42 | 6.67 | 19.95 | 0.37 | 0.33 |  |  | 49 | 36 | 1.14 | 7.59 |  | 0.17 | 17 | 0.21 | 0.03 |  |  |
|  | 39 | 7.11 | 19.94 | 0.67 |  |  |  | 49 | 29 | 2.44 | 5.78 |  |  | 14 | 0.22 | 0.07 |  |  |
|  | 51 | 2.51 | 17.325 | 1.67 | 0.5 | 0.17 | 0.83 | 50 | 44 | 2.54 | 5.92 | 0.54 |  | 16 | 0.23 | 0.06 |  | 0.03 |
|  | 44 | 6.79 | 19.95 | 0.81 | 0.22 |  |  | 50 | 39 | 2.83 | 6.585 |  |  | 16 | 0.25 | 0.03 | 0.03 |  |
|  | 47 | 6.53 | 21.47 | 0.51 |  |  |  | 51 | 34 | 5.1 | 6.18 | 0.18 |  | 18 | 0.2 | 0.04 |  |  |
|  | 54 | 7.83 | 18.745 | 1.17 | 0.33 | 0.17 |  | 51 | 45 | 2.21 | 6.41 | 0.27 | 0.54 | 18 | 0.19 | 0.03 |  | 0.03 |
|  | 66 | 5.17 | 20.635 | 1.52 |  |  |  | 51 | 36 | 3.66 | 4.68 | 0.66 |  | 15 | 0.22 | 0.04 |  |  |
|  | 67 | 6.83 | 20.705 | 0.64 | 0.21 |  |  | 51 | 47 | 1.97 | 5.835 | 0.12 |  | 14 | 0.23 | 0.04 | 0.03 |  |
|  | 59 | 6.5 | 21.25 | 0.5 | 0.125 |  |  | 51 | 41 | 3.18 | 4.57 |  | 0.17 | 13 | 0.19 | 0.03 | 0.03 |  |
|  | 62 | 12.17 | 14.26 | 1.1 | 0.54 | 1.17 |  | 52 | 51 | 3.1 | 7.45 |  |  | 18 | 0.19 | 0.02 |  | 0.05 |
|  | 81 | 4.33 | 22.59 | 0.83 |  |  |  | 52 | 49 | 2.94 | 6.55 | 0.32 |  | 17 | 0.18 | 0.03 |  |  |
|  | 40 | 4.39 | 23.32 | 0.31 | 0.01 |  |  | 52 | 29 | 0.86 | 7.57 |  |  | 16 | 0.17 | 0.04 | 0.03 |  |
|  | 47 | 9.54 | 20.915 | 0.21 |  |  |  | 52 | 37 | 0.34 | 6.83 |  |  | 14 | 0.15 | 0.03 |  |  |
|  | 80 | 8.25 | 21.625 | 0.5 |  |  |  | 53 | 48 | 1.54 | 7.23 |  |  | 16 | 0.16 | 0.02 |  |  |
|  | 66 | 6.75 | 19.875 | 1.5 | 0.5 |  |  | 53 | 36 | 5.47 | 3.91 | 0.57 |  | 15 | 0.3 | 0.03 | 0.03 |  |
|  | 70 | 7.34 | 20.215 | 0.93 | 0.61 |  |  | 53 | 50 | 3.29 | 4.465 | 0.46 | 0.1 | 14 | 0.25 | 0.02 |  |  |
|  | 62 | 6.91 | 21.18 | 0.83 | 0.31 |  |  | 53 | 49 | 1.27 | 6.78 | 0.43 | 0.22 | 17 | 0.18 | 0.03 | 0.02 |  |
|  | 67 | 7.75 | 21.5 | 0.75 | 0.25 |  |  | 54 | 45 | 2.86 | 6.57 |  |  | 16 | 0.17 | 0.03 | 0.04 |  |
|  | 59 | 4.75 | 24.25 | 0.25 |  |  |  | 54 | 39 | 2.21 | 6.185 | 0.14 |  | 15 | 0.18 | 0.02 |  |  |
|  | 49 | 5.72 | 23.465 | 0.45 |  |  |  | 54 | 40 | 3.4 | 6.03 | 0.18 |  | 16 | 0.18 | 0.05 |  |  |
|  | 50 | 6.09 | 20.93 | 0.86 | 0.43 | 0.35 |  | 54 | 39 | 1.95 | 5.215 | 0.54 |  | 14 | 0.15 | 0.03 | 0.02 | 0.04 |
|  | 61 | 16.01 | 18.66 | 0.33 | 0.17 |  |  | 55 | 48 | 6.96 | 3.985 | 0.21 | 0.11 | 16 | 0.15 | 0.02 |  |  |
| mean value |  | 6.95 | 19.55 | 0.83 | 0.27 | 0.07 | 0.03 | 50.13 |  | 3.08 | 5.54 | 0.26 | 0.06 | 15.16 | 0.21 | 0.03 | 0.01 | 0.01 |
| standard deviations |  | 2.55 | 3.00 | 0.58 | 0.31 | 0.22 | 0.15 | 3.48 |  | 1.57 | 1.79 | 0.27 | 0.12 | 2.57 | 0.04 | 0.01 | 0.01 | 0.01 |
| coefficient of variation |  | 36.71 | 15.33 | 69.21 | 115.35 | 317.79 | 556.78 | 6.94 |  | 50.98 | 32.30 | 107.28 | 210.10 | 16.95 | 20.93 | 36.82 | 142.39 | 237.10 |
| P |  | <0.01 | <0.01 | <0.01 | <0.01 | <0.01 | <0.01 | <0.01 |  | <0.01 | <0.01 | <0.01 | <0.01 | <0.01 | <0.01 | <0.01 | <0.01 | <0.01 |

**Table S3 Chromosome configuration of PMC MI in wheat-*E. elongata* BC_2_F_1_ hybrids**

| Lines | Observed Cells No. | Average chromosome configurations | | | | | | Chromosome No. | Observed Cells No. | Average Chromosome configurations of *E. elongata* | | | | *E. elongata* chromosome No. | Average Chromosome configurations of wheat-*E. elongata* | | | |
| --- | --- | --- | --- | --- | --- | --- | --- | --- | --- | --- | --- | --- | --- | --- | --- | --- | --- | --- |
|  |  | Ⅰ | Ⅱ | Ⅲ | Ⅳ | Ⅴ | Ⅵ |  |  | Ⅰ | Ⅱ | Ⅲ | Ⅳ |  | Ⅱ | Ⅲ | Ⅳ | V |
| BC_2_F_1_ | 71 | 11.77 | 13.13 | 0.75 | 0.43 |  |  | 42 | 52 | 5.24 | 1.88 |  |  | 9 | 0.2 | 0.03 | 0.03 |  |
|  | 57 | 11.33 | 12.68 | 1.33 | 0.33 |  |  | 42 | 49 | 6.8 | 0.6 |  |  | 8 | 0.18 | 0.03 |  |  |
|  | 77 | 8.84 | 15.52 | 0.32 | 0.29 |  |  | 42 | 51 | 5.7 | 0.15 |  |  | 6 | 0.17 | 0.04 | 0.04 |  |
|  | 64 | 9.39 | 15.04 | 0.67 | 0.13 |  |  | 42 | 44 | 6.1 | 0.45 |  |  | 7 | 0.21 | 0.06 |  |  |
|  | 56 | 10.21 | 14.66 | 0.53 | 0.22 |  |  | 42 | 36 | 7.6 | 0.2 |  |  | 8 | 0.22 | 0.07 |  |  |
|  | 63 | 8.95 | 13.71 | 0.37 | 1.38 |  |  | 43 | 43 | 4.96 | 1.02 |  |  | 7 | 0.2 | 0.09 | 0.05 |  |
|  | 63 | 9.46 | 12.865 | 1.77 | 0.58 |  | 0.03 | 43 | 47 | 6.9 | 0.55 |  |  | 8 | 0.19 | 0.03 |  |  |
|  | 71 | 9.11 | 14.795 | 0.94 | 0.37 |  |  | 43 | 41 | 5.9 | 0.55 |  |  | 7 | 0.17 | 0.03 | 0.03 |  |
|  | 80 | 9.31 | 14.79 | 0.84 | 0.26 | 0.31 |  | 44 | 50 | 5.12 | 0.44 |  |  | 6 | 0.2 | 0.04 | 0.04 | 0.04 |
|  | 54 | 8.21 | 16.475 | 0.48 | 0.35 |  |  | 44 | 34 | 4.87 | 0.565 |  |  | 6 | 0.33 | 0.06 |  |  |
|  | 55 | 9.78 | 15.985 | 0.35 | 0.24 |  | 0.04 | 44 | 41 | 5.2 | 0.9 |  |  | 7 | 0.36 | 0.05 |  |  |
|  | 72 | 10.05 | 15.99 | 0.51 | 0.11 |  |  | 44 | 52 | 7.21 | 0.395 |  |  | 8 | 0.28 | 0.08 | 0.04 |  |
|  | 63 | 8.94 | 16.13 | 0.42 | 0.21 | 0.14 |  | 44 | 40 | 4.21 | 2.395 |  |  | 9 | 0.2 | 0.05 |  |  |
|  | 49 | 9.78 | 15.56 | 0.89 | 0.22 | 0.11 |  | 45 | 29 | 8.34 | 1.33 |  |  | 11 | 0.31 | 0.06 | 0.03 | 0.03 |
|  | 53 | 9.01 | 16 | 1.33 |  |  |  | 45 | 35 | 6.5 | 0.75 |  |  | 8 | 0.39 | 0.08 |  |  |
|  | 52 | 9.51 | 16.115 | 0.54 | 0.41 |  |  | 45 | 37 | 8.37 | 0.815 |  |  | 10 | 0.2 | 0.05 |  |  |
|  | 62 | 8.94 | 15.66 | 0.74 | 0.63 |  |  | 45 | 49 | 7.22 | 0.89 |  |  | 9 | 0.19 | 0.07 |  |  |
|  | 67 | 9.65 | 15.845 | 0.54 | 0.51 |  |  | 45 | 44 | 6.85 | 0.575 |  |  | 8 | 0.2 | 0.09 | 0.04 |  |
|  | 47 | 9.4 | 15.8 | 1.4 | 0.2 |  |  | 46 | 26 | 6.67 | 1.665 |  |  | 10 | 0.18 | 0.11 |  |  |
|  | 47 | 8.54 | 16.26 | 0.98 | 0.5 |  |  | 46 | 27 | 6.31 | 1.345 |  |  | 9 | 0.22 | 0.14 | 0.02 |  |
|  | 56 | 9.18 | 16.28 | 0.74 | 0.51 |  |  | 46 | 36 | 7.21 | 0.395 |  |  | 8 | 0.33 | 0.07 |  |  |
|  | 55 | 11 | 17.34 |  | 0.33 |  |  | 47 | 37 | 7.02 | 0.99 |  |  | 9 | 0.37 | 0.11 |  |  |
|  | 53 | 10.51 | 15.445 | 0.84 | 0.62 | 0.12 |  | 47 | 49 | 9.64 | 0.68 |  |  | 11 | 0.38 | 0.09 | 0.03 | 0.03 |
|  | 65 | 10.16 | 16.485 | 0.57 | 0.54 |  |  | 47 | 45 | 8.21 | 0.895 |  |  | 10 | 0.33 | 0.09 | 0.02 |  |
|  | 45 | 11.05 | 15.485 | 0.84 | 0.74 | 0.1 |  | 48 | 35 | 7.95 | 0.525 |  |  | 9 | 0.2 | 0.07 |  |  |
|  | 59 | 10.97 | 16.17 | 0.71 | 0.64 |  |  | 48 | 39 | 6.31 | 0.845 |  |  | 8 | 0.36 | 0.08 | 0.03 |  |
|  | 55 | 10.77 | 16.907 | 1.01 | 0.231 |  | 0.077 | 49 | 49 | 5.44 | 1.28 |  |  | 8 | 0.23 | 0.06 |  |  |
|  | 61 | 11.23 | 15.975 | 0.88 | 0.52 | 0.22 |  | 49 | 37 | 5.66 | 0.67 |  |  | 7 | 0.23 | 0.07 | 0.04 | 0.04 |
|  | 70 | 10.67 | 16.595 | 0.67 | 0.62 | 0.21 | 0.1 | 50 | 45 | 7.65 | 0.675 |  |  | 9 | 0.25 | 0.08 |  |  |
| mean value |  | 9.85 | 15.51 | 0.76 | 0.42 | 0.04 | 0.01 | 45.07 |  | 6.59 | 0.84 |  |  | 8.28 | 0.25 | 0.07 | 0.02 | 0.002 |
| standard deviations |  | 0.94 | 1.16 | 0.37 | 0.26 | 0.08 | 0.02 | 2.33 |  | 1.26 | 0.50 |  |  | 1.36 | 0.07 | 0.03 | 0.02 | 0.01 |
| coefficient of variation |  | 9.52 | 7.49 | 48.82 | 62.86 | 199.61 | 283.50 | 5.17 |  | 19.18 | 59.75 |  |  | 16.43 | 29.18 | 38.96 | 118.98 | 257.42 |
| P |  | <0.01 | <0.01 | <0.01 | <0.01 | <0.01 | <0.01 | <0.01 |  | <0.01 | <0.01 | <0.01 | <0.01 | <0.01 | <0.01 | <0.01 | <0.01 | <0.01 |
